# Amikacin Liposome Inhalation Suspension in the Real-World Management of Refractory Mycobacterium avium Complex Pulmonary Disease

**DOI:** 10.7759/cureus.56622

**Published:** 2024-03-21

**Authors:** Toyoshi Yanagihara, Hiroaki Ogata, Asami Mori, Masako Kadowaki, Yuki Moriuchi, Akiko Ishimatsu, Junji Otsuka, Kazuhito Taguchi, Atushi Moriwaki, Makoto Yoshida

**Affiliations:** 1 Respiratory Medicine, National Hospital Organization Fukuoka National Hospital, Fukuoka, JPN; 2 Pharmacy, National Hospital Organization Fukuoka National Hospital, Fukuoka, JPN; 3 Infectious Diseases, National Hospital Organization Fukuoka National Hospital, Fukuoka, JPN

**Keywords:** guideline-based therapy, refractory mac pulmonary disease, amikacin liposome inhalation suspension, nontuberculous mycobacteria (ntm), mycobacterium avium complex

## Abstract

The increasing prevalence of *Mycobacterium avium* complex (MAC) pulmonary disease poses a significant therapeutic challenge, particularly due to the limited efficacy and systemic toxicity associated with conventional guideline-based therapy. Amikacin liposome inhalation suspension (ALIS) has been developed, yet its real-world application remains underreported. This retrospective analysis, conducted from March 2021 to February 2024, examined ALIS's clinical use in patients aged 20 years or older with refractory MAC pulmonary disease at our institution. The primary objective of this study is to describe the patient characteristics and clinical trajectories associated with the initiation of ALIS therapy in real-world settings for individuals diagnosed with MAC pulmonary disease. Of 11 patients initiated on ALIS, one was excluded due to financial constraints impacting continuation. The analysis proceeded with the remaining 10 subjects. The mean age of participants was 70.2 years, with a predominance of female patients (n = 7, 70%) and a higher incidence of *M. avium* infections (n = 6, 60%). Forty percent of the cohort (n = 4) had a history of ethambutol-induced optic neuritis leading to the cessation of the drug. The average interval from the initiation of guideline-based therapy to the start of ALIS was 8.5 ± 6.9 years (mean ± standard deviation). The majority (80%) presented with positive Gaffky scores at ALIS initiation, and a significant proportion exhibited resistance to clarithromycin and ethambutol. Comorbid conditions, including diabetes and previous cancer, were noted. The study also observed elevated anti-MAC antibody levels. Treatment duration varied, with fatigue leading to discontinuation in two cases. Treatment-emergent adverse events were documented in individual patients, each presenting with grade 1 severity: hemoptysis (n = 1, 10%), elevated creatinine levels (n = 1, 10%), and dysphonia (n = 2, 20%) were observed, respectively. Correlation analysis revealed a significant inverse relationship between body mass index (BMI) and ALIS discontinuation due to fatigue, and a positive correlation between Gaffky scores and C-reactive protein (CRP) levels. These results underscore the potential benefits and limitations of ALIS, suggesting that timely intervention and comprehensive healthcare support are crucial for optimal outcomes in the treatment of advanced MAC pulmonary disease.

## Introduction

The prevalence of pulmonary disease caused by nontuberculous mycobacteria (NTM) is on the rise, with *Mycobacterium avium* complex (MAC) being the predominant cause [1]. Current guideline-based therapy (GBT) predominantly prescribes macrolide-based combination treatments, supplemented by injectable aminoglycosides for cases that are severe or exhibit macrolide resistance. Despite these treatments, only approximately 60% of patients suffering from MAC pulmonary disease attain successful outcomes as per these guidelines, indicating a considerable fraction faces ongoing illness [2,3]. Besides, the usefulness of these intravenous aminoglycosides is frequently restricted because of the possibility of systemic toxicities, particularly renal and auditory toxicities. amikacin liposome inhalation suspension (ALIS), an innovative aerosolized form of amikacin encapsulated within liposomes, has been developed [4]. This advanced formulation facilitates precise amikacin delivery directly to the pulmonary system via aerosol nebulization, thereby increasing assimilation by alveolar macrophages and significantly reducing adverse systemic reactions [5,6]. The CONVERT study, which examined the efficacy of ALIS in managing treatment-resistant MAC pulmonary disease, demonstrated a marked improvement in culture conversion rates post-ALIS treatment [7]. Consequently, current guidelines recommend the use of ALIS for patients with MAC pulmonary disease who fail to exhibit culture conversion following a minimum of six months of standard therapeutic intervention [8]. In Japan, despite ALIS being covered by national health insurance since 2021, its high cost and the necessity for a specific inhalation system restrict its widespread application. This study aims to share our clinical insights and experiences employing ALIS in the management of patients with refractory MAC pulmonary disease within the context of real-world clinical practice, highlighting both its potential and the challenges faced in broader implementation.

## Materials and methods

Study participants

This study was conducted on a selected cohort of patients with MAC pulmonary disease treated with ALIS, aged 20 years or older, at our institution. Patients eligible for inclusion in this study were those who matched the criteria established by the American Thoracic Society and the Infectious Diseases Society of America (ATS/IDSA) guideline, necessitating the fulfillment of clinical and microbiological benchmarks: (i) respiratory symptoms linked to multifocal bronchiectasis and numerous small nodules evident on CT scans; (ii) thorough exclusion of alternative diagnoses; and (iii) confirmation of NTM infection through positive culture findings from a minimum of two distinct expectorated sputum specimens or a positive culture from at least one bronchial wash or lavage [9]. The timeframe for this retrospective analysis spanned from March 1, 2021, to February 29, 2024. A thorough examination of existing medical records was performed to extract relevant clinical information. The Institutional Review Board provided ethical approval for this research (Approval Number F5-35), which was conducted in accordance with the principles of the Helsinki Declaration. Owing to the study's retrospective design, the requirement for informed consent was waived. Patient data were anonymized to maintain the confidentiality and privacy of the participants. In each case, pulmonologists examined the patient's health records and collected data encompassing clinical, radiological, and microbiological aspects, as well as treatment-emergent adverse events (TEAEs). The severity of TEAEs was determined by the Common Terminology Criteria for Adverse Events (CTCAE): grade 1 (mild), grade 2 (moderate), grade 3 (severe), grade 4 (life-threatening), and grade 5 (death). Minimal inhibitory concentrations (MICs) were determined through the broth microdilution technique for drug susceptibility testing. The MAC antibody concentration was measured using the enzyme-linked immunosorbent assay at SRL, Inc. (Tokyo, Japan).

Statistical analyses

All results are presented as the mean, standard deviation (SD), and numbers (percentages) describing each variable. The statistical examination of the correlation matrix for ALIS discontinuation, age, sex, body mass index (BMI), Gaffky at ALIS initiation, AMK MIC, MAC antibody titer, C-reactive protein (CRP), and hemoglobin was executed utilizing Pearson correlation coefficients, with the analyses performed via GraphPad Prism version 10.

## Results

Baseline characteristics of the study subjects

Within the study period, 11 patients initiated ALIS therapy. However, one participant withdrew in two weeks for financial reasons and was thus excluded from the analysis. The remaining cohort comprised ten patients, as delineated in Table 1.

**Table 1:** Clinical profiles and treatment details of patients with MAC pulmonary disease initiating ALIS therapy. The table enumerates individual cases, detailing patient age, sex, body mass index (BMI), and prior treatment regimens, including azithromycin (AZM), clarithromycin (CAM), rifampicin (RFP), sitafloxacin (STFX), ethambutol (EB), and intravenous amikacin (AMK) where indicated. The duration from the initiation of guideline-based therapy (GBT) to the start of ALIS treatment is expressed in years. The Gaffky score at the commencement of ALIS treatment is provided for each case. Drug susceptibility testing results for CAM, AMK MIC, EB, and RFP are presented, with susceptibility categorized as sensitive (S), intermediate (I), or resistant (R). Cases where data are not available are marked with an asterisk (*).

Cases	Age	Sex	BMI	Treatment	Time from GBT to ALIS (yrs)	Gaffky at ALIS initiation	CAM	AMK MIC	EB	RFP
1	61	F	17.8	AZM+RFP+STFX+AMK	8	5	R	4	R	S
2	79	M	17.2	CAM+RFP+STFX	9.2	5	S	8	R	I
3	82	F	17.1	CAM+RFP+EB	1.3	2	S	8	R	I
4	44	F	20.8	CAM+RFP+EB	0.8	0	*	*	*	*
5	70	F	12.3	CAM+RFP+STFX	3.4	2	S	2	I	S
6	72	F	17.3	CAM+RFP+STFX+AMK	3.2	0	R	1	R	S
7	76	F	16.8	CAM+RFP+EB+STFX	18	2	S	4	R	S
8	76	M	20.4	AZM+RFP+STFX	10.5	3	R	16	R	S
9	75	F	19.5	CAM+RFP+EB	21.3	2	R	4	R	I
10	67	M	16.1	AZM+RFP+EB+STFX	9.3	4	S	2	R	S

**Figure 1:**
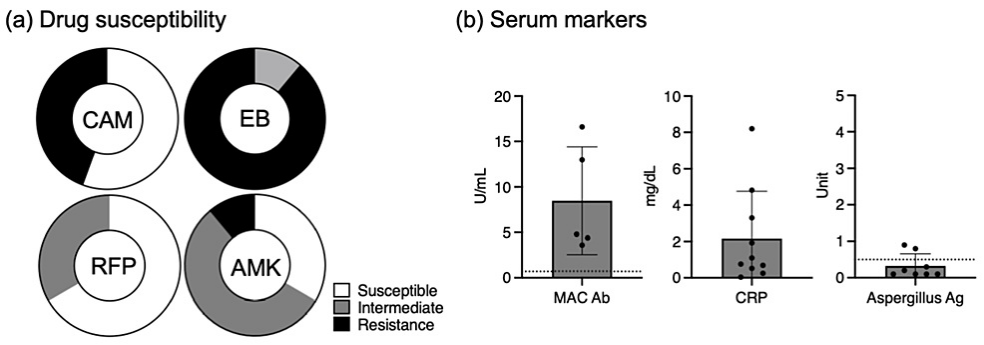
Clinical information of the patients in the cohort study. (a) Drug susceptibility: Pie charts representing the susceptibility profiles of *Mycobacterium avium* complex (MAC) to different antibiotics. Clarithromycin (CAM), ethambutol (EB), rifampicin (RFP), and amikacin (AMK) are depicted, with sections indicating the proportion of isolates that are susceptible (white), have intermediate susceptibility (light gray), or are resistant (dark gray) to each drug. (b) The serum concentration of anti-MAC antibodies (MAC Ab), C-reactive protein (CRP), and Aspergillus antigen (Aspergillus Ag) titers are shown. Individual data points are plotted over the box plots, which show the mean with SD. Outliers denote reference cutoffs.

The cohort's mean age was 70.2 ± 11.0 years, with females constituting 70% of the sample. The mean body mass index (BMI) was 17.5 ± 2.5 kg/m^2^. The distribution of the infecting organism was 60% *M. avium* and 40% *M. intracellulare*. The average interval from the initiation of guideline-based therapy (GBT) to the start of ALIS was 8.5 ± 6.9 years (mean ± SD), with 80% of cases exhibiting positive Gaffky scores at the onset of ALIS treatment. A notable 44.4% of the patients harbored clarithromycin-resistant strains (4/9), while 89% exhibited resistance to ethambutol (8/9) (Figure 1A). Amikacin resistance (≥16 μg/mL) was observed in a minority of the cases (1/9), and no rifampicin resistance was detected. 40% of the cohort had a history of ethambutol-induced optic neuritis leading to the cessation of the drug. Comorbid conditions were present in several cases, including type 2 diabetes mellitus (2 cases), systemic sclerosis, and Sjögren syndrome treated with 3.5 mg of prednisone (1 case), and a history of cancer (breast and stomach, 1 case each). All evaluated patients exhibited pronounced elevations in anti-MAC antibody concentrations (5/5), averaging 8.48 ± 5.92 U/mL-markedly exceeding the established clinical reference point of 0.7 U/mL (Figure 1B). C-reactive protein (CRP) levels varied among patients, with a range from 0.04 to 8.20 mg/dL. The detection of serum Aspergillus antigen was mostly below the clinical significance cutoff (6/8), with two instances slightly above the threshold (0.8 and 0.9 units), considered to be of minimal clinical concern.

**Figure 2:**
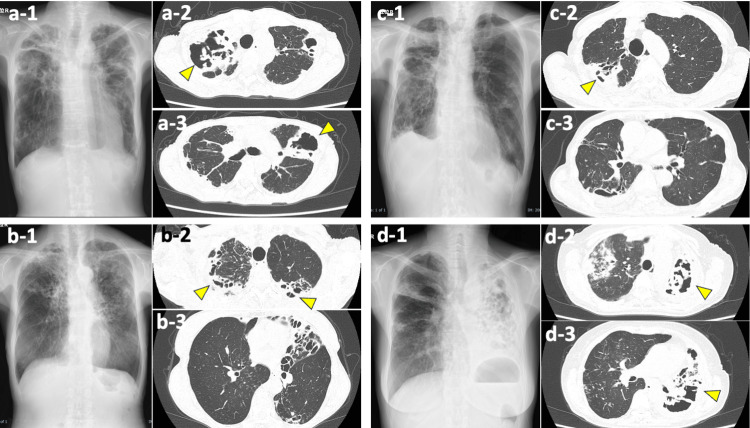
Radiographic Profiles of Pulmonary Manifestations. (a-1), (b-1), (c-1), and (d-1) depict the anterior-posterior chest radiographs, while (a-2,3), (b-2,3), (c-2,3), and (d-2,3) display the axial computed tomography (CT) scans of four distinct patients, denoted as (a), (b), (c), and (d), respectively. Arrowheads in the images highlight the presence of cavitary lesions within the pulmonary parenchyma.

All patients presented with cavitary pulmonary lesions (Figure 2).

Timeline of ALIS treatment and reasons for discontinuation in patients with MAC infections

**Figure 3:**
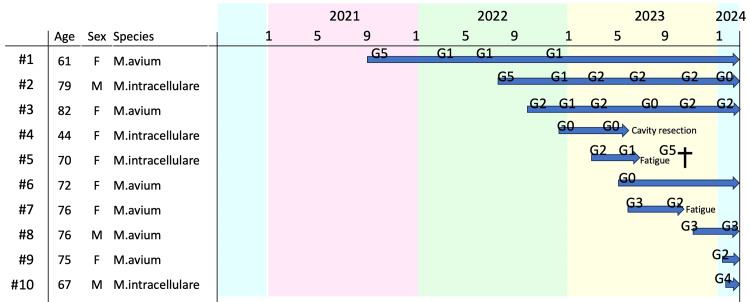
Timeline of ALIS treatment and reasons for discontinuation in patients with MAC pulmonary disease. Displayed is a timeline detailing the administration of amikacin liposome inhalation suspension (ALIS) to ten patients (#1 to #10), characterized by age, sex, and the *Mycobacterium avium* complex (MAC) identified (*M. avium* or *M. intracellulare*). The treatment period, spanning from 2021 to Feb 2024, is represented by horizontal arrows, with specific months noted above. Key events leading to discontinuation of ALIS are indicated: for patient #4, treatment cessation due to surgical resection of the infected cavity; for patient #5, discontinuation because of fatigue subsequent to a COVID-19 infection; and for patient #7, treatment was halted owing to fatigue. Each event is symbolized by an arrow pointing to the date of ALIS discontinuation for the respective patients. "G5" on the timeline refers to the Gaffky score 5 at a specific time point.

The ALIS treatment duration, as depicted in Figure 3, varied among participants due to the retrospective study design. Notably, the follow-up duration was under six months for three patients post-ALIS initiation. Discontinuation of ALIS was prompted by specific events: surgical removal of an infected cavity for patient #4; post-COVID-19 fatigue leading to cessation after three months for patient #5; and fatigue after four months of treatment for patient #7. For Patient #5, the fatigue experienced was classified as grade 3 severity, with persistent lethargy necessitating bed rest throughout the day. Regarding Patient #7, the medical records did not yield sufficient information to accurately assign a fatigue severity grade. Hemoptysis (grade 1) and transient increases in creatinine levels (grade 1) were observed in one patient each, necessitating a temporary halt in ALIS administration. Two patients experienced dysphonia (grade 1) without discontinuation of ALIS. The Gaffky score positivity was noted in most cases following ALIS initiation, and the short follow-up period impeded adequate assessment of culture conversion.

Correlation of the clinical parameters

**Figure 4:**
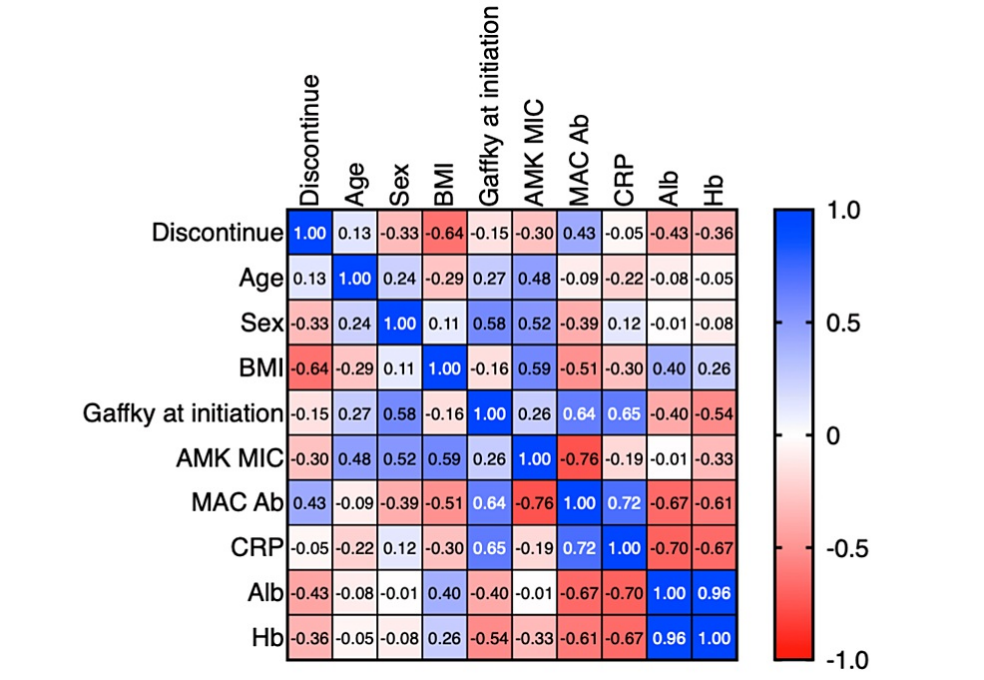
A heatmap displaying the correlation coefficients between various clinical and laboratory parameters. Positive correlations are indicated in shades of blue, negative correlations in shades of red, and the scale ranges from -1.0 to 1.0. Parameters include discontinuation of treatment (discontinue), patient age, sex, body mass index (BMI), Gaffky scale at ALIS treatment initiation (Gaffky at initiation), amikacin minimum inhibitory concentration (AMK MIC), MAC antibody levels (MAC Ab), CRP, albumin (Alb), and hemoglobin (Hb). The strength of the correlation is directly proportional to the intensity of the color.

Last, we assessed the interrelationships between clinical parameters as illustrated in the correlation matrix (Figure 4). ALIS treatment was terminated in two instances due to fatigue. Our findings identified a significant inverse correlation between BMI and the cessation of ALIS due to fatigue (r = -0.641, p = 0.046). Additionally, a robust positive correlation was observed between Gaffky scores and C-reactive protein (CRP) levels (r = 0.653, p = 0.041). While not reaching statistical significance, there was an observable trend suggesting an association of anti-MAC antibody titers with both Gaffky scores and CRP levels, as well as with the MIC of amikacin. An inverse relationship was evident between CRP levels and both albumin and hemoglobin concentrations, indicative of the inflammatory state's impact.

## Discussion

In our study, we delineated the demographic and clinical attributes of patients with refractory MAC pulmonary diseases commencing ALIS therapy. Limited literature exists regarding ALIS treatment in real-world practice, with one report from South Korea involving six cases [10]. The mean age of our cohort was elevated compared to that in the CONVERT study (70.2 vs 64.7) [7]. Furthermore, the average BMI was substantially lower in our cohort than that reported in the CONVERT study (17.5 vs 21.2 kg/m^2^), aligning with trends observed in the Japanese subgroup analysis from the same study, where the mean BMI was also reduced compared to the broader cohort (19.1 vs 21.2 kg/m^2^) [11]. This may hint at potential ethnic variances in disease presentation or body composition. Remarkably, even within the Japanese subgroup, our cohort's BMI was lower, implying the inclusion of more clinically advanced cases in real-world ALIS initiation. Our correlation analysis revealed a significant association between lower body mass index (BMI) and the discontinuation of ALIS due to fatigue, reinforcing the premise that patients with advanced disease may exhibit decreased tolerance to ALIS therapy. This finding emphasizes the necessity for timely intervention with ALIS to enhance treatment sustainability and patient outcomes.

The clarithromycin resistance rate within our cohort exceeded that reported in both the CONVERT study and its Japanese subgroup (44.4% vs 21.8% vs 35.4%, respectively), and an overwhelming majority exhibited resistance to ethambutol (89%). These findings suggest that the prolonged duration of GBT prior to the introduction of ALIS may contribute to the emergence of drug resistance. The increased resistance underscores the need for alternative therapeutic options like ALIS. Nevertheless, the lower resistance rate to amikacin observed in our cohort underscores a compelling rationale for the initiation of ALIS therapy. This resistance profile highlights the potential of ALIS as a strategic treatment in the management of refractory MAC pulmonary diseases, particularly in the context of existing macrolide and ethambutol resistance.

Within our cohort, the rate of culture conversion was not accurately determined due to the limited duration of follow-up. However, among the cases with follow-up periods exceeding one year, a trend of diminishing Gaffky scores was observed, suggesting an improvement in disease severity over time. In the Japanese subgroup analysis of the CONVERT study, the proportion of Japanese patients in the ALIS + GBT group achieved 26.5% culture conversion by Month 6, which was comparable to that observed in the overall population (29.0%). Given the higher incidence of clarithromycin and ethambutol-resistant MAC in this cohort, ALIS initiation is expected to improve disease progression. The optimal duration for ALIS treatment remains undefined within the current medical literature. Notably, the INS-312 study, a 12-month open-label extension trial, observed ALIS administration extending up to 20 months [12]. The safety profile and tolerability of ALIS in combination with GBT during the INS-312 extension were in alignment with findings from the CONVERT study, with no new safety concerns emerging from up to 20 months of ALIS exposure. This evidence suggests a potential for extended ALIS use, although further research is required to establish definitive treatment duration guidelines.

In terms of the GBT regimen, it was noted that 30% of the cases in our cohort incorporated azithromycin. International guidelines advocate for azithromycin-based treatments over those based on clarithromycin, citing improved tolerability, fewer drug interactions, and the convenience of a single daily dose, without compromising efficacy [8]. In the context of Japan, azithromycin was not approved for insurance coverage until 2020, and there is an insurance guideline-recommended preference to consider clarithromycin as the initial therapeutic option. This practice may contribute to the lower utilization rate of azithromycin. However, it is anticipated that the selection of azithromycin will increase in the future, aligning with international guidelines and emerging clinical preferences.

Fifty percent of our patients ceased ethambutol due to optic neuritis; yet, none progressed to blindness, maintaining visual integrity. The incidence of ethambutol-induced optic neuropathy in our cohort was significantly higher than the generally reported rate of 0.44% [13]. This discrepancy suggests that the cohort under study had a longer treatment period and regular vision screenings allowed for the early identification of optic neuropathy. Furthermore, research by Morimoto et al. has demonstrated a clear link between ethambutol cessation during GBT and an increased likelihood of developing clarithromycin resistance [14]. This association highlights the necessity of careful monitoring during GBT and the potential need to revise treatment strategies in patients with a history of ethambutol use.

In our cohort, all patients demonstrated marked increases in anti-MAC antibody levels, with an average concentration of 8.48 U/mL, significantly exceeding the clinical threshold. Shibata et al. conducted a systematic review and meta-analysis involving 1,098 cases of pulmonary MAC disease and 2,270 controls across 16 selected studies [15]. When focusing on 14 studies utilizing commercially available MAC antibody kits, the estimated sensitivity was 69.6% and specificity was 90.6%, yielding a positive likelihood ratio of 7.4 and a negative likelihood ratio of 0.34 with the cutoff value set at 0.7 U/mL [15]. These findings suggest that anti-MAC antibodies possess favorable diagnostic characteristics for MAC disease. Moreover, levels of anti-MAC antibodies have been correlated with both disease activity and therapeutic responses in patients with MAC lung disease [16,17]. Thus, measuring anti-MAC antibody titers could be advantageous for the clinical management of patients with MAC lung disease.

In our cohort, all 10 patients who commenced ALIS treatment demonstrated good adherence. A significant factor contributing to this adherence appears to be the high cost of ALIS. Indeed, there were reports from patients expressing a sentiment along the lines of, "Since the medication is expensive, I must make a concerted effort to continue with it." The process of cleaning, disinfecting, and assembling the nebulizer components is indeed time-consuming and initially posed concerns for the patients. However, after a week-long hospital stay, patients were able to learn and master the entire procedure.

The primary limitation of our study is the short period of follow-up after ALIS initiation, limited case numbers, and a retrospective design. Consequently, the findings concerning the correlation between the cessation of ALIS and other clinical parameters, as well as the incidence and severity of TEAEs, are provisional. The main purpose of this study was to elucidate the characteristics of refractory MAC infections that necessitate ALIS induction in a real-world context. While providing valuable insights, the results should be viewed as exploratory. Further research, incorporating longer follow-up, larger patient samples, and prospective designs, is essential to validate and elaborate on our findings.

## Conclusions

Our investigation revealed that ALIS therapy was initiated in clinical practice for patients exhibiting a high rate of drug resistance with a generally favorable safety profile. However, the discontinuation of ALIS in cases complicated by fatigue suggests that advanced disease states may diminish tolerance to the treatment. Timely intervention with comprehensive healthcare support is crucial for optimal outcomes in the treatment of advanced MAC pulmonary disease.

## References

[1] Prevots DR, Marras TK (2015). Epidemiology of human pulmonary infection with nontuberculous mycobacteria: a review. Clin Chest Med.

[2] Diel R, Nienhaus A, Ringshausen FC, Richter E, Welte T, Rabe KF, Loddenkemper R (2018). Microbiologic outcome of interventions against Mycobacterium avium complex pulmonary disease: a systematic review. Chest.

[3] Kwak N, Park J, Kim E, Lee CH, Han SK, Yim JJ (2017). Treatment outcomes of Mycobacterium avium complex lung disease: a systematic review and meta-analysis. Clin Infect Dis.

[4] Meers P, Neville M, Malinin V (2008). Biofilm penetration, triggered release and in vivo activity of inhaled liposomal amikacin in chronic Pseudomonas aeruginosa lung infections. J Antimicrob Chemother.

[5] Zhang J, Leifer F, Rose S (2018). Amikacin liposome inhalation suspension (ALIS) penetrates non-tuberculous mycobacterial biofilms and enhances amikacin uptake into macrophages. Front Microbiol.

[6] Olivier KN, Griffith DE, Eagle G (2017). Randomized trial of liposomal amikacin for inhalation in nontuberculous mycobacterial lung disease. Am J Respir Crit Care Med.

[7] Griffith DE, Eagle G, Thomson R (2018). Amikacin liposome inhalation suspension for treatment-refractory lung disease caused by Mycobacterium avium complex (convert). A prospective, open-label, randomized study. Am J Respir Crit Care Med.

[8] Daley CL, Iaccarino JM, Lange C (2020). Treatment of nontuberculous mycobacterial pulmonary disease: an official ATS/ERS/ESCMID/IDSA clinical practice guideline. Clin Infect Dis.

[9] Griffith DE, Aksamit T, Brown-Elliott BA (2007). An official ATS/IDSA statement: diagnosis, treatment, and prevention of nontuberculous mycobacterial diseases. Am J Respir Crit Care Med.

[10] Kim BG, Kim SR, Jhun BW (2023). Real-world outcomes of amikacin liposome inhalation suspension for refractory Mycobacterium avium complex pulmonary disease. Tuberc Respir Dis (Seoul).

[11] Morimoto K, Nonaka M, Yamazaki Y (2024). Amikacin liposome inhalation suspension for Mycobacterium avium complex pulmonary disease: a subgroup analysis of Japanese patients in the randomized, phase 3, CONVERT study. Respir Investig.

[12] Winthrop KL, Flume PA, Thomson R (2021). Amikacin liposome inhalation suspension for Mycobacterium avium complex lung disease: a 12-month open-label extension clinical trial. Ann Am Thorac Soc.

[13] Masataka M, Masamitsu H, Toshiyasu S (2013). An incidence of the ethambutol-induced optic neuropathy and the usefulness of a periodic visual acuity test: a questionnaire survey in Japan. Ann Japanese Respir Soc.

[14] Morimoto K, Namkoong H, Hasegawa N (2016). Macrolide-resistant Mycobacterium avium complex lung disease: analysis of 102 consecutive cases. Ann Am Thorac Soc.

[15] Shibata Y, Horita N, Yamamoto M (2016). Diagnostic test accuracy of anti-glycopeptidolipid-core IgA antibodies for Mycobacterium avium complex pulmonary disease: systematic review and meta-analysis. Sci Rep.

[16] Fukushima K, Kitada S, Matsumoto Y (2021). Serum GPL core antibody levels are associated with disease activity and treatment outcomes in Mycobacterium avium complex lung disease following first line antibiotic treatment. Respir Med.

[17] Ogata H, Moriwaki A, Nakagawa T (2021). Association of serum antibodies against the Mycobacterium avium complex and hemoptysis: a cross-sectional study. BMC Infect Dis.

